# The ultralow viscosity of volatile-rich kimberlite magma: Implications for the water content of primitive kimberlite melts

**DOI:** 10.1126/sciadv.ado8550

**Published:** 2024-09-06

**Authors:** Ming Hao, Wen-Yi Zhou, Rostislav Hrubiak, Curtis Kenney-Benson, Janine L. Kavanagh, William Davis, Jin S. Zhang

**Affiliations:** ^1^ Department of Earth and Planetary Sciences, University of New Mexico, Albuquerque, NM, USA.; ^2^ Earth and Planets Laboratory, Carnegie Institution for Science, Washington, DC, USA.; ^3^ Department of Geology and Geophysics, Texas A&M University, College Station, TX, USA.; ^4^ HPCAT, Advanced Photon Source, Argonne National Laboratory, Argonne, IL, USA.; ^5^ School of Environmental Sciences, University of Liverpool, Liverpool L69 3GP, UK.; ^6^ Cecil H. and Ida M. Green Institute of Geophysics and Planetary Physics, Scripps Institution of Oceanography, University of California, San Diego, La Jolla, CA 92037, USA.

## Abstract

The eruption of deeply sourced kimberlite magma offers the fastest route to bring deep-seated volatiles back to the Earth’s surface. However, the viscosity of kimberlite magma, a factor governing its migration and eruption dynamics within Earth, remains poorly constrained. We conducted synchrotron in situ falling sphere viscometry experiments to examine kimberlite magma with different volatile contents (0 to 5 wt % H_2_O and 2 to 8 wt % CO_2_) under high pressure-temperature conditions. The results reveal that the viscosity of volatile-rich kimberlite magma is ~1 to 2 orders lower than that of mid-ocean ridge basalt (MORB) and comparable to the ultramobile pure carbonate melt. Using the measured viscosity values, we simulated the ascent and eruption process of kimberlite magma. We found that a minimum content of ~0.5 wt % water in the primitive magma is necessary to allow the ultrafast eruption process of kimberlite, thereby enabling the preservation of diamonds and high-pressure mineral inclusions transported by the magma.

## Teaser

Kimberlite magmas with over 0.5 wt % water ascend rapidly due to their extremely low viscosity.

## INTRODUCTION

Kimberlite magma, known for hosting diamonds, holds substantial importance among terrestrial magmas owing to its volatile-rich composition and deep-seated origin (*1*–*4*) (e.g., >200 km). They are excellent geochemical probes of the deep Earth (*5*, *6*). For example, the Mg/Si ratios of kimberlite magma and the mantle xenoliths enclosed within it can help to constrain the local mantle chemical composition (*5*). Typically, kimberlite magma originates from partial melting of CO_2_-bearing peridotite (*7*–*9*) or subducted carbonated slab crust (*10*, *11*) at depths greater than ~200 km, possibly within the mantle transition zone (*2*, *11*, *12*). Therefore, kimberlite serves as an important portal into understanding the deep volatile cycles inside Earth (*6*). Nevertheless, the water content of primitive kimberlite magma remains controversial, highlighted by recent conflicting studies (*5*, *13*). The water in primitive kimberlite magma not only influences its transport properties but also affects the elemental partitioning (*14*). Moreover, if the source mantle of kimberlite is wet, then it could serve as an indicator for ancient subduction zones (*15*). Therefore, it is critical to constrain the water content in the primitive kimberlite melts.

Determining the water content of primitive kimberlite melts using the collected field samples is difficult, partially due to post-emplacement alteration processes such as serpentinization, potential surface water additions at shallower depths, and interactions between the kimberlite magma and host rocks during dike ascent (*16*–*18*). For example, Russell *et al.* (*16*, *17*) suggested that during ascent, primitive kimberlite magma with a carbonate melt phase entraps mantle xenoliths and assimilates pyroxenes within these xenoliths, which leans to the increase in SiO_2_ content of the melt and the release of CO_2_, forming the observed typical kimberlite at the surface. The inferred water content of primitive kimberlite from field samples varies from <1 wt % to 6 to 9 wt % (*19*–*21*). Thus, exploring aspects beyond traditional rock sampling could offer valuable insights into the elusive water content of primitive kimberlite melts.

Viscosity is an important physical property influencing the migration and eruption dynamics of magmas. Kimberlite magma is renowned for its remarkable ascent and eruption speed (*1*, *16*, *22*). Sparks *et al.* (*22*) modeled kimberlite magma eruptions and suggested that a sufficiently low viscosity would enable the extremely rapid eruption process (e.g., ~4 to 20 m/s) under the turbulent flow regime. As an important network modifier, H_2_O is expected to have strong influence on the viscosity of magmas (*23*–*25*). Hence, an exploration into the volatile-dependent viscosity of kimberlite magmas holds the potential to provide additional constraints on the water content of primitive kimberlite melts, offering insights into their rapid eruption processes.

However, the viscosity of volatile-rich kimberlite magma, especially regarding the influence of water content, lacks robust experimental constraints. A recent study using quenched falling sphere experiments found that the viscosity of kimberlite magma is comparable to or even higher than that of MORB under high pressure-temperature conditions and does not depend on the H_2_O content (*26*). Such high viscosity values do not support the fast ascent of kimberlite magma proposed in previous studies (*1*, *22*).

To address this knowledge gap, we conducted in situ falling sphere viscometry measurements on kimberlite magma with 2 to 8 wt % CO_2_ and 0 to 5 wt % H_2_O up to 5.3 GPa and 2173 K using the Paris-Edinburgh press at Sector 16-BM-B, High-Pressure Collaborative Access Team (HPCAT), Advanced Photon Source (*27*) and examined the chemical composition of a representative run product that survived after decompression (Materials and Methods, figs. S1 and S3, and table S1). Then, we calculated the viscosity of kimberlite magma under different pressure-temperature conditions using the terminal velocities, which are the calculated maximum sphere falling speeds via analyzing x-ray images from a high-speed camera. Using the new viscosity data in this study, we further model the ascent and eruption processes of kimberlite magma and discuss the possible range of water content in primitive kimberlite melts.

## RESULTS

**Fig. 1.**
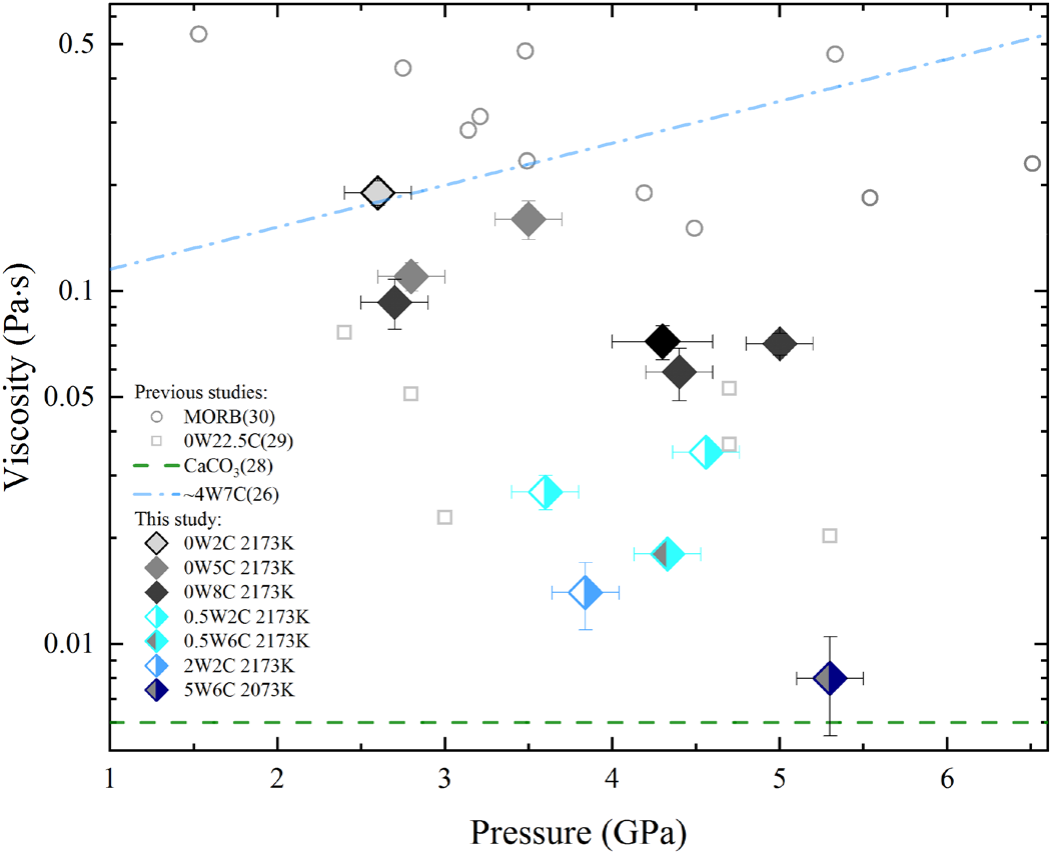
The viscosities of kimberlite, MORB, CaCO3, and carbonate-silicate transitional melts under high pressure-temperature conditions. The numbers before the “W” and “C” represent the wt % of the H_2_O and CO_2_ in the melts, respectively. The diamond symbols represent the viscosity values of kimberlite magmas with different CO_2_ and H_2_O contents from this study. The gray circles and squares represent the viscosity measurements of MORB (*30*) and carbonate-silicate transitional melts with ~22.5 wt % CO_2_ (*29*), respectively. The blue dash-dotted line represents the viscosity values of kimberlite magma from the quenched experiments (*26*). The green dashed line represents the viscosity of pure carbonate melts (*28*).

Figure 1 shows all the experimental results. With only 0.5 wt % H_2_O, the viscosity of kimberlite magma decreases by a factor of ~6, from 0.1 to 0.2 Pa·s to 0.02 to 0.03 Pa·s. With 2 wt % H_2_O, the viscosity of kimberlite magma decreases by more than an order of magnitude. When the H_2_O content reaches 5 wt %, the viscosity of kimberlite magma drops to less than 0.01 Pa·s, approaching values akin to that of pure carbonate melts (*28*) at similar pressure-temperature conditions and on the same order of the viscosity of liquid water at ambient condition. Addition of CO_2_ could also decrease the viscosity of kimberlite magma, consistent with prior studies reporting ultralow viscosity values for pure carbonate and carbonate-silicate transitional melts (*28*, *29*). However, our results underscore the stronger impact of H_2_O compared to CO_2_. For example, with addition of only 0.5 wt % H_2_O and 2 wt % CO_2_, the viscosity of kimberlite magma closely resembles that of anhydrous carbonate-silicate transitional melts with ~22.5 wt % CO_2_ (*29*).

**Fig. 2.**
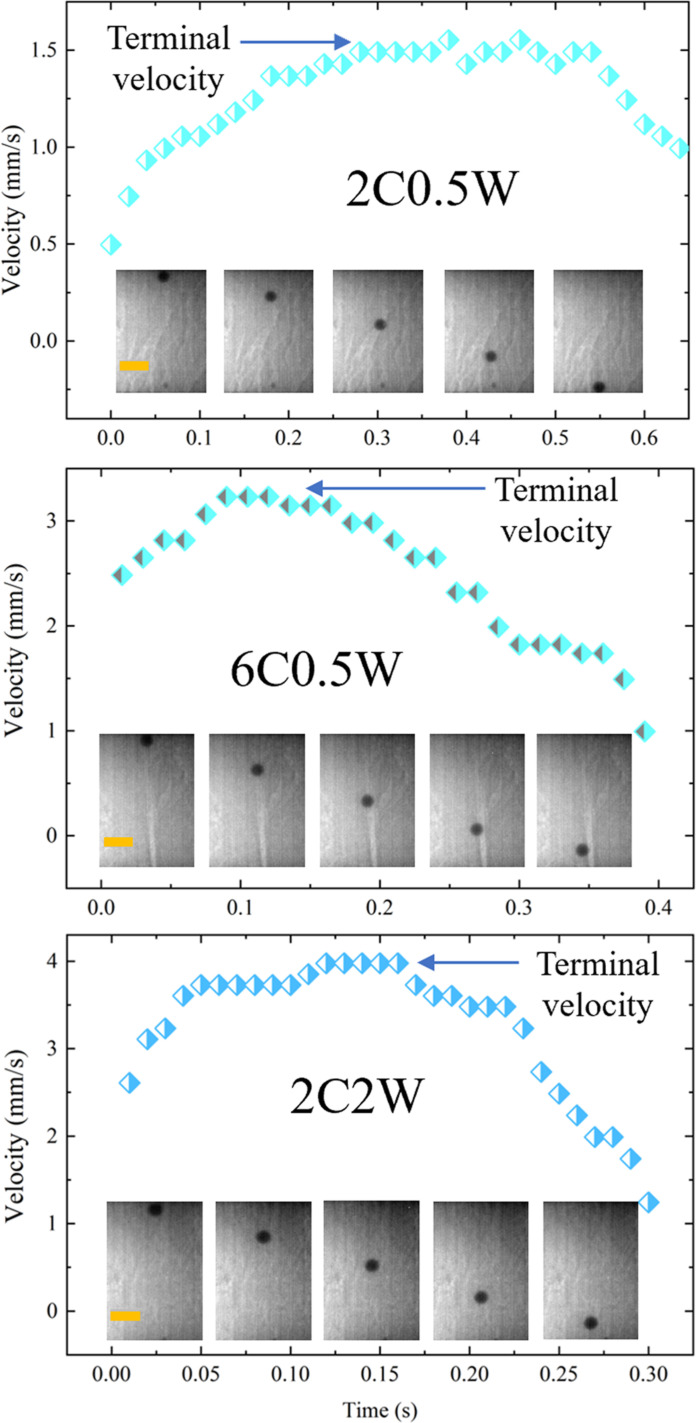
Movements of the rhenium spheres and the velocity evolution during the falling sphere viscosity measurements. The numbers before the W and C represent the wt % of the H_2_O and CO_2_ in the melts, respectively. All spheres reached terminal velocities during the experiments. The orange bars in the images represent 0.2 mm.

Persikov *et al.* (*26*) is the only existing experimental study regarding the viscosity of hydrous kimberlite magma at high pressure-temperature conditions. The reported viscosity values in Persikov *et al.* (*26*) for compositions similar to those in our study are ~0.1 to 0.7 Pa·s which are comparable to the measured viscosity of MORB at similar pressure-temperature conditions using in situ falling sphere viscometry (*30*). The reported viscosity of hydrous kimberlite magma in Persikov *et al.* (*26*) is even higher than MORB at pressures higher than ~3.5 GPa. However, our experimental data, which also used in situ falling sphere viscometry, reveals that the viscosity values of kimberlite magma with different volatile contents are always lower than MORB across the investigated pressure-temperature range (~2 to 5 GPa and 2073 to 2173 K). The notable higher viscosity values in Persikov *et al.* (*26*) may result from the lower velocities of falling spheres using the quenched method. In the quenched experiments, the sphere falling process was not directly recorded, leading to the determination of average sphere falling speeds instead of terminal velocity (Fig. 2). Consequently, the calculated viscosity values using the average sphere falling speeds in their quenched experiments likely represent upper limits rather than the true viscosity values of the liquids. In contrast, as shown in Fig. 2, we can calculate the velocity change over time throughout the entire sphere falling process using x-ray radiography images, which enables us to accurately determine the terminal velocities (*31*).

## DISCUSSION

**Fig. 3.**
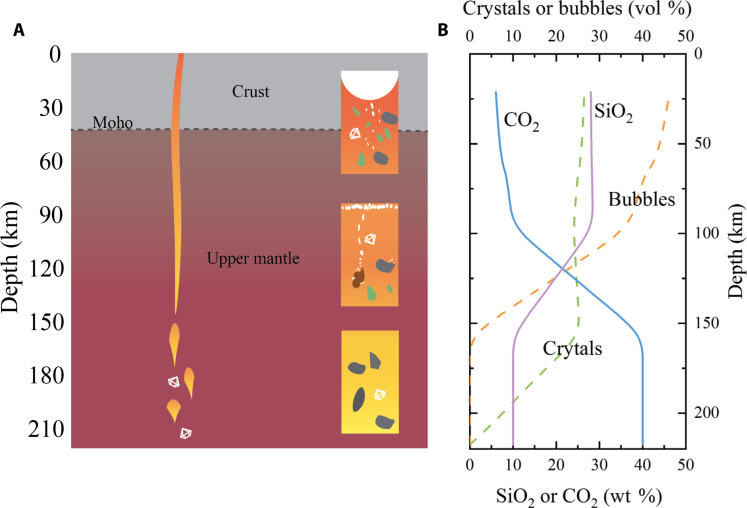
Kimberlite magma ascent and evolution process. (**A**) Schematic illustration of the kimberlite magma ascent and eruption process. (**B**) The calculated compositional changes of kimberlite magma with depth. The CO_2_ and SiO_2_ contents represent the chemical variations of the melts. The crystal content shows the total volume fractions in the magma. The bubble content includes both the bubble within the magma and accumulated at the tip of the dyke.

To investigate the influence of water on the ascent and eruption process of kimberlite magma, we calculated the chemical composition, xenoliths content, and bubble content of kimberlite magma change as a function of depth, drawing from established models by Sparks *et al.* (*22*) and Rusell *et al.* (*16*, *17*) (Fig. 3). As shown in Fig. 3, the original kimberlite magma is carbonate melt at depth greater than 150 km. At the initial stage of ascent, the original carbonate melt gradually assimilates mantle xenoliths with limited chemical reactions, and thus only the proportion of suspended crystals increases (*17*). At ~100- to 150-km depth, the pyroxenes, especially orthopyroxene, within the captured mantle xenoliths react with carbonate melts (*16*), releasing CO_2_. This released CO_2_ undergoes a bubbling process, potentially separating from the remaining melts due to the low density of CO_2_ fluids and the reduced viscosity of the melts. Consequently, a volatile-rich buoyant tip emerges at this stage. Despite this, the overall crystal content remains relatively stable due to the countereffects of xenoliths assimilation and simultaneous chemical reaction. As xenoliths dissolve in the melts, the CO_2_ content in the melts decreases, while the SiO_2_ content increases. At depth less than 100 km, the ongoing release of CO_2_ results primarily from pressure exsolution instead of chemical reactions. In addition, olivine crystals start to crystallize within this depth range. The detailed calculation of the chemical composition evolution for the primitive kimberlite at depths is shown in Materials and Method.

The formation of crystals and bubbles resulting from assimilation and exsolution can substantially influence the effective viscosity of magmas (text S1) (*32*). As shown in Fig. 3, the released bubbles could either suspend within the melts or accumulate forming a volatile-rich tip. Since only the bubbles suspended in the melts affect the effective viscosity, we examined three different scenarios to calculate the viscosity of primitive kimberlite melts during ascent and eruption: (i) CO_2_ bubbles completely separate from the melt, exerting no influence on the effective viscosity; (ii) all CO_2_ bubbles remain suspended in the melt without any segregation, thereby excluding the formation of a volatile-rich tip; (iii) only a small amount of newly released CO_2_ bubbles (~5 vol %) remains suspended in the melts, while the majority segregates from the melt, forming a volatile-rich tip. Then, using the rheology of the three-phase suspension model with a high capillary number (text S1), we calculated the effects of crystals and bubbles on the viscosity of primitive kimberlite melts (*32*). To cover the large composition range of the kimberlite magma, we have included both the experimental results from this study and data from Stagno *et al.* (*29*) on carbonate-silicate transitional melts to model the effects of CO_2_ and H_2_O on the viscosity of kimberlite melts. In addition, we corrected for temperature effects using the predicted temperature profile outlined by Kavanagh and Sparks (*33*). The equations and detailed calculations are shown in text S1.

**Fig. 4.**
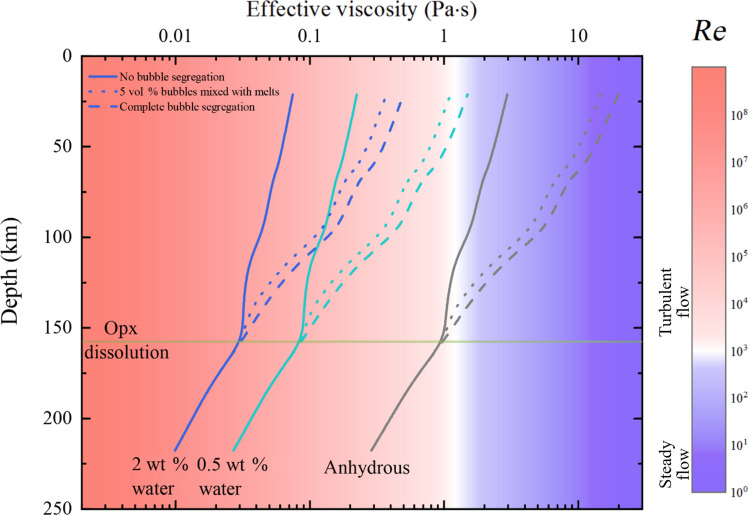
Modeled effective viscosity of primitive kimberlite melts changes as a function of depth. The bubbles start to show up at depth of ~150 km where orthopyroxene (opx) starts to react with carbonate melts. A *Re* number larger than 1000 represents turbulent flow, and a *Re* number smaller than 10 represents steady flow. The horizontal axe is in log scale.

Turbulent flow regime has been suggested for kimberlite magma eruption based on petrologic textural evidence of field kimberlite samples (e.g., the milling of xenoliths and xenocrysts in kimberlite deposits) (*34*, *35*). The ultrafast eruption of kimberlite magma, as indicated by various eruption models, also requires adoption of the turbulent flow regime (*17*, *22*). The critical Reynolds number (*Re*) to form turbulent kimberlite magma flows during eruption is ~1000, corresponding to an effective viscosity of 1.24 Pa·s (*22*). As shown in Fig. 4, we assumed three different water contents (anhydrous, 0.5 and 2 wt %) in primitive kimberlite magma and fixed the water content at different depths to estimate the effective viscosity of kimberlite melts during the ascent and eruption. The calculated viscosity profiles at three hydration levels do not necessarily reflect the actual water content variations during its ascent but provided useful insights to the range of viscosity values we would expect during the eruption (*33*). At depths greater than ~150 km, because of the low viscosity of carbonate melts (*28*), the primitive kimberlite magmas with mantle xenoliths are all in turbulent flow regime regardless of the water content. At depths between 50 and 150 km, the exsolution of CO_2_ in primitive kimberlite melts gradually alters the chemical composition from carbonate to typical ultramafic silicate kimberlite melts (*17*). The effective viscosity gradually increases, but the hydrous kimberlite melts are still well within the turbulent flow regime. However, the effective viscosity of anhydrous kimberlite magmas is high enough causing the corresponding *Re* number to decrease below 1000, placing the flow regime between turbulent and steady flow. At depth less than 50 km, the continuous exsolution of CO_2_ and olivine crystallization further elevates the effective viscosity of kimberlite melts. With 0.5 wt % water in the melts, the *Re* number is still close to 1000 even with complete CO_2_ bubble segregation, while the *Re* number for anhydrous kimberlite is already approaching or even falling below 10 with partially segregated bubbles.

Considering the natural eruption process of kimberlite magma, a volatile-rich tip is expected (*1*, *17*). Thus, partial or complete bubble segregation is a more realistic scenario. In this case, anhydrous primitive kimberlite melts exhibit excessive viscosity at the top of upper mantle, hindering turbulent flow regimes. However, the addition of only 0.5 wt % water is enough to maintain turbulent flow in the magma. Therefore, a minimal quantity of water is essential for the fast ascent and eruption of primitive kimberlite magma in the upper mantle.

To summarize, we used falling sphere viscometry to measure the viscosity of volatile-rich kimberlite magma, revealing viscosity values 1 to 2 orders of magnitude lower than previous studies (*26*). On the basis of these viscosity measurements, we quantified the effect of water on the ascent and eruption dynamics of kimberlite magma. Our findings indicate that a relatively small quantity of water (~0.5 wt %) plays a crucial role in enabling the rapid ascent and eruption of primitive kimberlite magma, which could help to preserve the high-pressure mineral phases (e.g., ringwoodite) in the super-deep diamonds (*36*, *37*) as well as the major elements, trace elements, and isotope signatures of the local mantle sampled by the xenoliths (*38*).

## MATERIALS AND METHODS

### Starting materials

It is difficult to determine a representative major element composition of kimberlite melt, since its chemical composition likely changes during its ascent process (*16*–*18*, *22*), from carbonate melts at depths greater than 150 km to carbonate-silicate transitional melts and lastly the observed ultramafic carbon-bearing kimberlite melts (*16*, *17*). Since previous studies has measured the viscosity of carbonate melts and carbonate-silicate transitional melts using in situ falling sphere viscometry (*28*, *29*), this study focuses on the ultramafic kimberlite melts with relatively low CO_2_ contents (2 to 8 wt % CO_2_) at different hydration levels (0 to 5 wt % H_2_O). The major element composition of the starting material is the average of the kimberlite field samples from nine different places (table S1). Oxides, carbonates, and hydroxides with purity > 99% were ground, mixed, and pressed as starting materials (*19*).

### Falling sphere viscometry

We conducted high pressure-temperature in situ falling sphere experiments at Sector 16-BM-B, HPCAT, Advanced Photon Source using Paris-Edinburgh cell in graphite capsules (*27*) with 1.5 mm in diameter and 2.0 mm in height (fig. S1). The temperatures were calculated from the power curve, which was calibrated according to the temperature at the center of the sample (*27*). The temperature uncertainty of each experimental run is estimated to be 100 K (*27*). The temperature gradient across the entire sample along the horizontal and vertical directions is <40 K at 2000 K (*39*), and thus the effect of temperature gradient on the obtained terminal velocity is minimal. The pressures were determined using the equation of state of MgO, with the pressure difference between MgO sleeve and the actual sample chamber taken into account (*27*).

For all experimental runs, once the target pressure was reached, we firstly increased temperature gradually from 300 to 1073 K, which is well below the solidus of the sample, in roughly 10 min. Then, we increased temperature abruptly from 1073 to 2073 K or 2173 K by adjusting the input power to the targeted value. The fast and complete melting was confirmed by immediate and straight descent of rhenium spheres after the power adjustment. Because of the ultrafast nature of these falling sphere experiments, volatile loss was negligible, and chemical contamination from graphite capsule was limited to the edge of the sample, as evidenced by the chemical analysis on the run product (*40*).

Parallel beam polychromatic x-rays were used for imaging the sample chamber (Fig. 2). A high-speed camera (Photron FASTCAM SA3) with 1000 frames per second was used to capture the sample chamber images during the sphere falling process. A tungsten carbide sphere with a 497-μm diameter was used to calibrate the size of the pixels of the camera. Rhenium spheres with diameters of ~80 to 190 μm were used to do falling sphere viscometry measurements. The solubility of rhenium is <10 parts per million in silicate melts at experimental conditions (*41*), and thus the sample is unlikely to be contaminated by rhenium spheres. The position of the rhenium spheres recorded by the high-speed camera was analyzed using ImageJ software to calculate sphere falling speeds. The terminal velocities (ν), which are the maximum sphere falling speeds, are calculated from the velocity-time curves (Fig. 2). The viscosity (η) was calculated with the Stokes equation with correction factors outlined in Kono *et al.* (*27*)

$$\eta =\frac{gd_{s}^{2}\left(\rho_{s}-\rho_{l}\right)F}{18\nu E}$$ (1)

$$F=1-2.104\left(\frac{d_{s}}{d_{l}}\right)+2.09{\left(\frac{d_{s}}{d_{l}}\right)}^{3}-0.95{\left(\frac{d_{s}}{d_{l}}\right)}^{5}$$ (2)

$$E=1+\frac{9d_{s}}{16Z}+{\left(\frac{9d_{s}}{16Z}\right)}^{2}$$ (3)

where *g* is gravitational acceleration, and *d* and ρ are diameters and densities of the sphere (*s*) and liquid (*l*), respectively. *Z* is the height of the sample capsule. *F* is the Faxén correction factor (*42*), and *E* is the end correction factor (*43*). Equation 1 is applicable for *d_s_*/*d_l_* ≪ 1 and *Re* ≪ 1. The first condition was satisfied by the experimental design, whereas post hoc validations of *Re* were attained by finding self-consistent solutions of the relation

$$\text{Re}=\frac{\rho_{l}d_{s}\nu }{\eta }$$ (4)

giving *Re* < 0.2. Uncertainties in viscosity estimates are largely influenced by the uncertainty in the terminal velocity measurements (*44*); alternative edge effect corrections gave viscosity estimates within uncertainties (*45*).

### Chemical analysis on quenched sample

Although most samples did not survive during the fast quench and decompression process, we were able to identify a well-preserved sample (#3-10 in table S2) which went through complete melting at 3.8 GPa 2173 K and contained ~2 wt % water and ~2 wt % of CO_2_. We then performed electron microprobe analysis (EPMA), energy-dispersive spectroscopy (EDS) mapping, and Fourier transform infrared spectra (FTIR) mapping to examine the chemical composition of the quenched sample as well as its homogeneity. The FTIR measurements were conducted using a Bruker HYPERION II FTIR microscope with a liquid N_2_ cooled mid-band mercury cadmium telluride detector at the Texas A&M University (*46*). The EPMA experiments were completed using a JEOL 8530F electron microprobe with an accelerating voltage of 15 kV and beam current of 30 nA at the Earth and Planets Laboratory, Carnegie Institute of Science. Wavelength dispersive spectroscopy was performed to quantify the chemical compositions with focused (~1 μm) and defocused beam (~5 μm) on random spots (table S1), and EDS mapping was performed to examine the chemical homogeneity of the entire sample (fig. S3). Experimental results are included in table S1 and fig. S3.

### The composition evolution of kimberlite melts at depth

According to previous experiments and models (*16*, *17*), the primary kimberlite melts at depth greater than 200 km are carbonate melts with ~10 wt % SiO_2_. During its ascent, at depth greater than 100 km, the melts gradually assimilate ~51 vol % orthopyroxene-rich mantle xenoliths (*47*, *48*) to reach a typical SiO_2_ content of ~30 wt %. At 150- to 100-km depth, the orthopyroxenes in xenoliths react with the melt, gradually releasing CO_2_ and the CO_2_ content in the melts decreases to ~10 wt %. At depth less than 100 km, the CO_2_ content continues to decrease, reaching a level of around 5 wt % due to the pressure-induced exsolution. At the same time, ~5 wt % olivine crystallizes from the melts. The composition evolution is displayed in Fig. 3.

### Calculation of *Re* number of kimberlite melts

The *Re* number for kimberlite melts in dyke can be calculated using (*22*)

$$\text{Re}=\frac{2\Delta P\rho w^{3}}{3h\mu^{2}}$$ (5)

where Δ*P* is the overpressure, *w* is the half dyke width, *u* is the velocity, ρ is the magma density, *h* is the dyke vertical length, and μ is the viscosity. Using typical values of Δ*P* = 10 MPa, *w* = 0.25 m, ρ = 3000 kg/m^3^, and *h* = 200 km, the *Re* number is ~1000 for a viscosity value of 1.24 Pa·s and the *Re* number is ~10 for a viscosity value of 12.4 Pa·s (*22*).

## Supplementary Materials

### This PDF file includes:

Supplementary Text S1 Figs. S1 to S3 Tables S1 and S2 References
