# Supplementary material for: The ultralow viscosity of volatile-rich kimberlite magma: Implications for the water content of primitive kimberlite melts
Source: Sci Adv. 2024 Sep 6;10(36):eado8550. doi: 10.1126/sciadv.ado8550 (PMC11378914; doi:10.1126/sciadv.ado8550)
Supplement: Supplementary file 1 — Supplementary Text S1 Figs. S1 to S3 Tables S1 and S2 References [file sciadv.ado8550_sm.pdf]

Science Advances
AAAS

Supplementary Materials for

The ultralow viscosity of volatile-rich kimberlite magma: Implications for the water content of primitive kimberlite melts

Ming Hao *et al.*

Corresponding author: Ming Hao, unmminghao@gmail.com; Jin S. Zhang, jinzhang@tamu.edu

Sci. Adv. **10**, eado8550 (2024)
DOI: 10.1126/sciadv.ado8550

This PDF file includes:

Supplementary Text S1
Figs. S1 to S3
Tables S1 and S2
References

Supplementary Text

Text S1. Calculation of the (effective) viscosity during magma ascent and eruption process

Assuming the viscosity of all silicate melts at infinite temperature is $10^{-4.6}$ Pa·s, the temperature effects of the viscosity were calculated by fitting the equation(*25*, *49*):

$$log\eta = 1000b/T - 4.6 \quad (1)$$

where T is temperature and b is a numerical constant, which can be fitted by the experimental results. According to our results and previous studies(*29*), pressure has subtle influences on the viscosity of kimberlite magma. Therefore, we did not consider the pressure effects in the model presented in this study.

During the kimberlite magma eruption process, the exsolved volatile bubbles and crystals also affect the effective viscosity (η_e). Based on the observed thickness of kimberlite dykes (~1 m) and the ascent rates (> 4-16 m/s), the strain rate for kimberlite magma is also very high (> 4-16 s^{-1})(*22*). Thus, the capillary number (*Ca*) for kimberlite is likely high and the η_e at high-*Ca* can be estimated using(*32*):

$$\eta_e = \eta_0(1-\phi_b)^{\frac{5}{3}}(1-\frac{\phi_c}{\phi_m})^{-2} \quad (2)$$

$$\phi_m = \phi_{m1}\exp\left(-\frac{(\log_{10} r_p)^2}{2b^2}\right) \quad (3)$$

where η_0 is the viscosity of the pure liquid, ϕ_m is the maximum packing fraction, r_p is the particle aspect ratio, ϕ_{m1} and b are empirically determined constant, and ϕ_c and ϕ_b are the volume fractions of the crystals and bubbles, respectively. Considering that the observed olivine crystals in kimberlite are well-rounded and the r_p varies from 1 to 2(*50*), the ϕ_{m1}, b, and r_p are fixed to be 0.66, 1.08, and 1.5, respectively. The ϕ_c and ϕ_b are calculated using:

$$\phi_c = \frac{V_c}{V_1 + V_b + V_c} \tag{4}$$

$$\phi_b = \frac{V_b}{V_1 + V_b} \tag{5}$$

where V_1, V_b, and V_c are the volumes of the melts, bubble, and crystal, respectively.

Utilizing the data presented in Stagno et al.(*29*) and this study, the effects of CO_2 content on the viscosity of anhydrous kimberlite magma (η_a) at 2173 K are fitted to the empirical equation:

$$\eta_a = 0.2744x^{-0.547} \tag{6}$$

where x is the weight percentage of the CO_2. For the effects of H_2O content (0-5 wt.%) on the viscosity of magma at 2173 K, the fitting based on the data in this study to empirical equation yields:

$$\eta = \frac{\eta_a}{11.624\ln(y+1) + 1.07} \tag{7}$$

where y is the weight percentage of H_2O.

We first utilized empirical equations (6) and (7) to calculate the viscosity of volatile-rich kimberlite magma at 2173 K. Then equation (1) is used for temperature correction. Equations (2-5) are used to calculate the final effective viscosity for bubble and crystal-bearing kimberlite magma when needed.

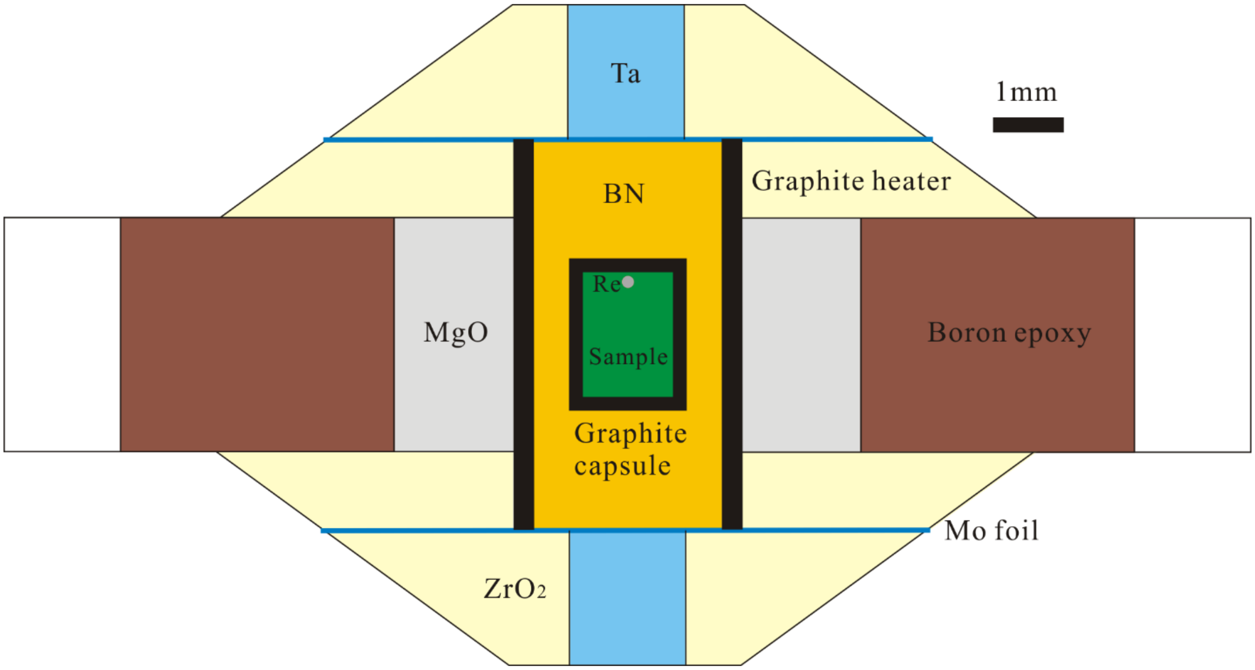

Fig. S1. Schematic illustration of the Paris-Edinburgh cell assembly used in this study(*28*).

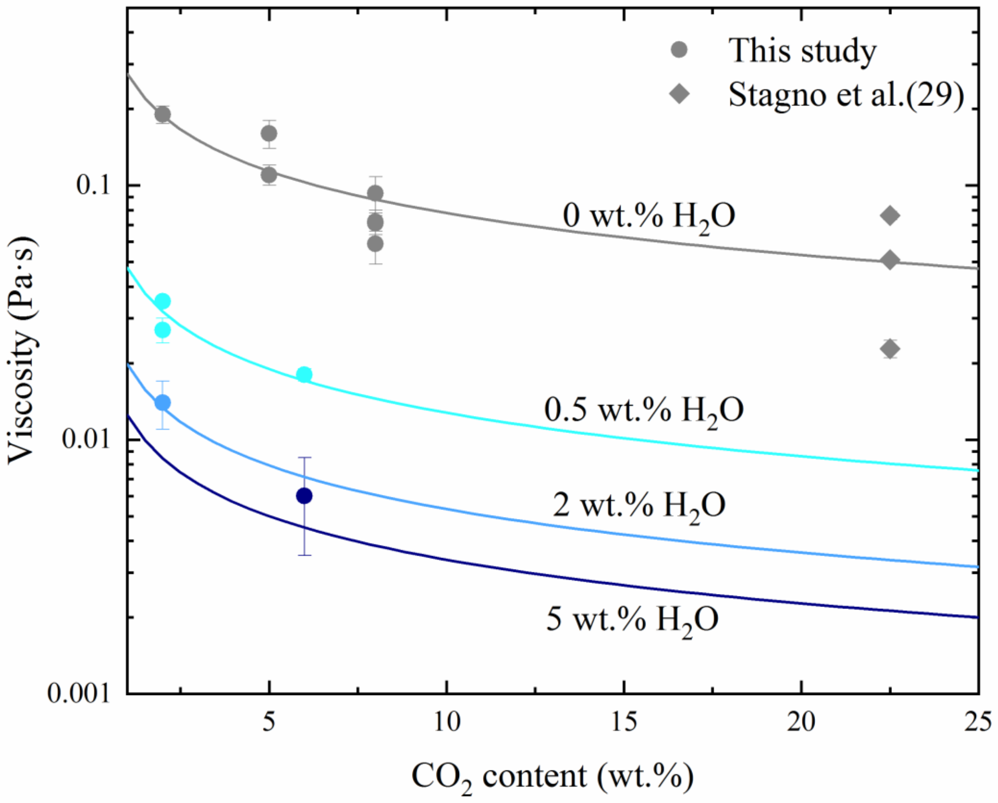

Fig. S2. CO_2 and H_2O dependent viscosity. Solid lines represent the fitting of actual experimental data presented in this study and Stagno et al.(*29*) (Equation (6) and (7) in Text S1).

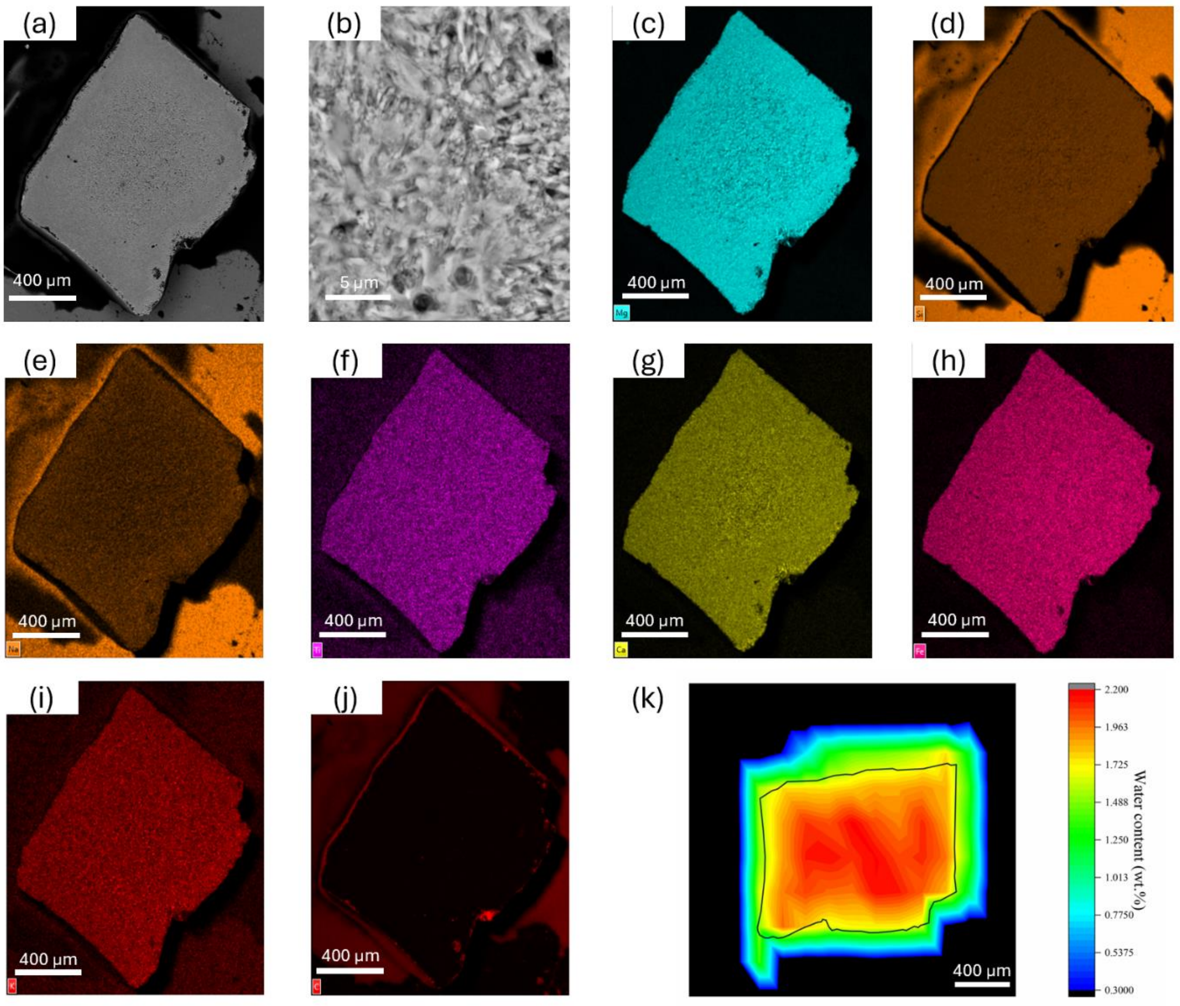

Fig. S3. Chemical analysis of the quenched sample (#3-10 in Table S2). (a) and (b) are Backscattered Electron images of the sample at two different scales. As expected, the ultramafic kimberlite melt was quenched into an ultra-fine-grained aggregate instead of a homogeneous glass. Slightly slower cooling at the center of the sample resulted in coarser grains observed in (b). (c)-(j) are EDS maps for Mg, Si, Na, Ti, Ca, Fe, K, and C, respectively. All the elements are homogeneously distributed across the sample. (k) is the water content map measured using FTIR, and the black line marks the edge of the sample. The larger water content gradient near the sample edge is due to the large spot size (~100 μm) used for FTIR mapping. The H_2O content was calculated using the calibration based on hydrous basalt(*41*), due to the lack of water content

calibration in kimberlite. The calculated H_2O content of 2.1(1) wt.% from FTIR measurements is in excellent agreement with 2 wt.% H_2O estimated from the starting materials, suggesting negligible loss of water during the experiments.

	South Africa(*20*)		Canada(*21*)		Greenland(*51*)		Russia(*19*)			Natural kimberlite sample composition Range(*19*)	This study
	kimberlite	primitive melts	kimberlite	primitive melts	kimberlite	primitive melts	low H_2O	high H_2O	primitive melts		
SiO_2	30.1-31.6	25.5-26.9	32.46	27.5-31.8	25.84	17.47	26.71	29.29	-	25.6-40	31.3[32.5]*
TiO_2	1.1-1.3	1.4-1.7	0.6	0.7-1.1	3.62	4.99	1.25	1.27	-	0.3-3.6	1.4[1.34]
Al_2O_3	1.2-1.4	1.1-1.3	2.01	2.5-3.1	1.42	2.27	1.75	1.79	-	1.4-3.0	2.1[1.81]
FeO	9.1-9.5	9.2-9.7	7.59	7.3-8.3	10.32	10.61	8.09	8.34	-	6.6-10.3	8.3[9.36]
MgO	31.2-33.8	25.4-27.6	34.28	27.5-30.8	33.36	23.98	31.33	33.14	-	25.2-38.6	32.5[32.6]
CaO	10.4-12.0	16.7-18.6	8.34	9.2-14.2	10.72	17.27	12.19	11.84	-	4.1-12.2	9.2[7.86]
Na_2O	0.2	0.2-0.3	0.07	0.1	0.09	0.13	3.23	0.31	6.2	0.1-3.2	0.52[0.28]
K_2O	0.8-1.0	0.7-0.9	0.72	0.6-1.0	0.2	0.32	1.33	1.01	-	0.2-1.5	0.96[0.54]
H_2O	2.3-2.7	3.0-3.5	6.54	6.1-8.7	-	-	0.38	2.84	<1	0.4-7.8	0-5
CO_2	6.8-8.3	9.3-11.2	6.07	5.1-12.2	-	-	9.42	7.96	-	2.6-9.4	2-8

*****The numbers in the bracket are the average chemical composition from EPMA measurements on randomly selected 10 spots of quenched sample #3-10. The accuracy of the EPMA measurements on this particular sample is limited due to the small mineral grain size, beam damage, and vaporization (in particular for those relatively volatile light elements). The values are normalized to share same total as the starting materials.

Table S1. The chemical compositions of different kimberlite samples and their corresponding primitive kimberlite melts. The last two columns show the composition range of natural kimberlite samples and composition of the samples used in this study. We adopted the average major element compositions of the kimberlite samples from different locations(*19*).

Run#	Composition	Pressure (psi)	Temperature (K)	Pressure (GPa)	Sphere diameter (mm)	Terminal Velocity (mm/s)	Viscosity (Pa·s)	Uncertainty (Pa·s)	Re
1-3	5W6C	7000	2073	5.3(2)	0.080(12)	7.1(3)	0.008	0.004	0.213
2-2	0W2C	5000	2173	2.6(2)	0.179(4)	1.00(5)	0.19	0.015	0.003
2-6	0W5C	4000	2173	2.8(2)	0.229(6)	2.3(1)	0.11	0.01	0.014
2-9	0W5C	5000	2173	3.5(2)	0.192(10)	1.30(5)	0.16	0.02	0.005
2-13	0W8C	4000	2173	2.7(2)	0.139(6)	1.40(5)	0.093	0.015	0.006
2-14	0W8C	5000	2123	4.4(2)	0.152(8)	2.5(1)	0.059	0.01	0.019
2-15	0W8C	5500	2173	4.3(3)	0.174(5)	2.5(1)	0.072	0.008	0.018
2-19	0W8C	5000	2173	5.0(2)	0.149(2)	2.0(1)	0.071	0.005	0.013
3-9	0.5W2C	5500	2173	4.6(2)	0.082(2)	1.50(5)	0.035	0.002	0.011
3-10	2W2C	5000	2173	3.8(2)	0.092(8)	4.5(2)	0.014	0.003	0.089
3-11	0.5W6C	5500	2173	4.3(2)	0.084(2)	3.1(1)	0.018	0.001	0.043
3-13	0.5W2C	4000	2173	3.6(2)	0.184(8)	7.1(2)	0.027	0.003	0.145

Table S2. The experimental results obtained in this study. The temperature uncertainty of each experimental run is <100 K. The density contrast between the liquids and spheres are estimated to be ~17.7 g/cm^3. The capsule dimensions from the quenched samples are fixed to be 1.1 mm in diameter and 1.1 mm in height. The uncertainties of the calculated viscosities are estimated to be ~10-20%, and mainly arise from the uncertainties of sphere diameter (~2%-5%), terminal velocity (~2%-5%), and the estimated density contrast between melts and spheres (<3%) due to the high-density contrast between the sphere and the melt.

REFERENCES AND NOTES

1. L. Wilson, J. W. Head III, An integrated model of kimberlite ascent and eruption. *Nature* **447**, 53–57 (2007).

2. S. F. Foley, G. M. Yaxley, B. A. Kjarsgaard, Kimberlites from source to surface: Insights from experiments. *Elements* **15**, 393–398 (2019).

3. K. G. Nickel, D. H. Green, Empirical geothermobarometry for garnet peridotites and implications for the nature of the lithosphere, kimberlites and diamonds. *Earth Planet. Sci. Lett.* **73**, 158–170 (1985).

4. R. H. Mitchell, *Kimberlites: Mineralogy, Geochemistry, and Petrology* (Springer Science & Business Media, 2013).

5. D. G. Pearson, J. Woodhead, P. E. Janney, Kimberlites as geochemical probes of Earth's mantle. *Elements* **15**, 387–392 (2019).

6. S. Tappe, R. L. Romer, A. Stracke, A. Steenfelt, K. A. Smart, K. Muehlenbachs, T. H. Torsvik, Sources and mobility of carbonate melts beneath cratons, with implications for deep carbon cycling, metasomatism and rift initiation. *Earth Planet. Sci. Lett.* **466**, 152–167 (2017).

7. R. Dasgupta, A. Mallik, K. Tsuno, A. C. Withers, G. Hirth, M. M. Hirschmann, Carbon-dioxide-rich silicate melt in the Earth's upper mantle. *Nature* **493**, 211–215 (2013).

8. M. Becker, A. P. L. Roex, Geochemistry of South African on- and off-craton, group I and group II kimberlites: Petrogenesis and source region evolution. *J. Petrol.* **47**, 673–703 (2006).

9. S. E. Price, J. K. Russell, M. G. Kopylova, Primitive magma from the Jericho Pipe, N.W.T., Canada: Constraints on primary kimberlite melt chemistry. *J. Petrol.* **41**, 789–808 (2000).

10. S. Tappe, D. Graham Pearson, B. A. Kjarsgaard, G. Nowell, D. Dowall, Mantle transition zone input to kimberlite magmatism near a subduction zone: Origin of anomalous Nd–Hf isotope systematics at Lac de Gras, Canada. *Earth Planet. Sci. Lett.* **371–372**, 235–251 (2013).

11. C. Sun, R. Dasgupta, Slab–mantle interaction, carbon transport, and kimberlite generation in the deep upper mantle. *Earth Planet. Sci. Lett.* **506**, 38–52 (2019).

12. E. S. Kiseeva, K. D. Litasov, G. M. Yaxley, E. Ohtani, V. S. Kamenetsky, Melting and phase relations of carbonated eclogite at 9–21 GPa and the Petrogenesis of Alkali-Rich melts in the deep mantle. *J. Petrol.* **54**, 1555–1583 (2013).

13. V. S. Kamenetsky, A. V. Golovin, R. Maas, A. Giuliani, M. B. Kamenetsky, Y. Weiss, Towards a new model for kimberlite petrogenesis: Evidence from unaltered kimberlites and mantle minerals. *Earth Sci. Rev.* **139**, 145–167 (2014).

14. A. G. Sokol, A. N. Kruk, Y. N. Palyanov, The role of water in generation of group II Kimberlite magmas: Constraints from multiple saturation experiments. *Am. Mineral.* **99**, 2292–2302 (2014).

15. S. Saha, R. Dasgupta, Phase relations of a depleted peridotite fluxed by a CO_2-H_2O fluid—Implications for the stability of partial melts versus volatile-bearing mineral phases in the cratonic mantle. *J. Geophys. Res. Solid Earth* **124**, 10089–10106 (2019).

16. J. K. Russell, L. A. Porritt, Y. Lavallée, D. B. Dingwell, Kimberlite ascent by assimilation-fuelled buoyancy. *Nature* **481**, 352–356 (2012).

17. J. K. Russell, R. S. J. Sparks, J. L. Kavanagh, Kimberlite volcanology: Transport, ascent, and eruption. *Elements* **15**, 405–410 (2019).

18. R. S. J. Sparks, Kimberlite volcanism. *Annu. Rev. Earth Planet. Sci.* **41**, 497–528 (2013).

19. V. S. Kamenetsky, M. B. Kamenetsky, Y. Weiss, O. Navon, T. F. Nielsen, T. P. Mernagh, How unique is the Udachnaya-East kimberlite? Comparison with kimberlites from the Slave Craton (Canada) and SW Greenland. *Lithos* **112**, 334–346 (2009).

20. A. Soltys, A. Giuliani, D. Phillips, A new approach to reconstructing the composition and evolution of kimberlite melts: A case study of the archetypal Bultfontein kimberlite (Kimberley, South Africa). *Lithos* **304-307**, 1–15 (2018).

21. B. A. Kjarsgaard, D. G. Pearson, S. Tappe, G. M. Nowell, D. P. Dowall, Geochemistry of hypabyssal kimberlites from Lac de Gras, Canada: Comparisons to a global database and applications to the parent magma problem. *Lithos* **112**, 236–248 (2009).

22. R. S. J. Sparks, L. Baker, R. J. Brown, M. Field, J. Schumacher, G. Stripp, A. Walters, Dynamical constraints on kimberlite volcanism. *J. Volcanol. Geotherm. Res.* **155**, 18–48 (2006).

23. K.-U. Hess, D. B. Dingwell, Viscosities of hydrous leucogranitic melts: A non-Arrhenian model. *Am. Mineral.* **81**, 1297–1300 (1996).

24. H. R. Shaw, Viscosities of magmatic silicate liquids: An empirical method of prediction. *Am. J. Sci.* **272**, 870–893 (1972).

25. D. Giordano, J. K. Russell, D. B. Dingwell, Viscosity of magmatic liquids: A model. *Earth Planet. Sci. Lett.* **271**, 123–134 (2008).

26. E. S. Persikov, P. G. Bukhtiyarov, A. G. Sokol, Viscosity of hydrous kimberlite and basaltic melts at high pressures. *Russ. Geol. Geophys.* **58**, 1093–1100 (2017).

27. Y. Kono, C. Park, C. Kenney-Benson, G. Shen, Y. Wang, Toward comprehensive studies of liquids at high pressures and high temperatures: Combined structure, elastic wave velocity, and viscosity measurements in the Paris–Edinburgh cell. *Phys. Earth Planet. In.* **228**, 269–280 (2014).

28. Y. Kono, C. Kenney-Benson, D. Hummer, H. Ohfuji, C. Park, G. Shen, Y. Wang, A. Kavner, C. E. Manning, Ultralow viscosity of carbonate melts at high pressures. *Nat. Commun.* **5**, 5091 (2014).

29. V. Stagno, Y. Kono, V. Stopponi, M. Masotta, P. Scarlato, C. E. Manning, "The viscosity of carbonate-silicate transitional melts at Earth's upper mantle pressures and temperatures, determined by the in situ falling-sphere technique" in *Carbon in Earth's Interior* (American Geophysical Union (AGU), 2020), pp. 223–236.

30. T. Sakamaki, A. Suzuki, E. Ohtani, H. Terasaki, S. Urakawa, Y. Katayama, K. Funakoshi, Y. Wang, J. W. Hernlund, M. D. Ballmer, Ponded melt at the boundary between the lithosphere and asthenosphere. *Nat. Geosci.* **6**, 1041–1044 (2013).

31. C. Liebske, B. Schmickler, H. Terasaki, B. Poe, A. Suzuki, K. Funakoshi, R. Ando, D. Rubie, Viscosity of peridotite liquid up to 13 GPa: Implications for magma ocean viscosities. *Earth Planet. Sci. Lett.* **240**, 589–604 (2005).

32. J. M. Truby, S. P. Mueller, E. W. Llewellin, H. M. Mader, The rheology of three-phase suspensions at low bubble capillary number. *Proc. R. Soc. A Math. Phys. Eng. Sci.* **471**, 20140557 (2015).

33. J. L. Kavanagh, R. S. J. Sparks, Temperature changes in ascending kimberlite magma. *Earth Planet. Sci. Lett.* **286**, 404–413 (2009).

34. R. C. Brett, J. K. Russell, G. D. M. Andrews, T. J. Jones, The ascent of kimberlite: Insights from olivine. *Earth Planet. Sci. Lett.* **424**, 119–131 (2015).

35. T. J. Jones, J. K. Russell, D. Sasse, Modification of mantle cargo by turbulent ascent of kimberlite. *Front. Earth Sci.* **7** (2019).

36. D. G. Pearson, F. E. Brenker, F. Nestola, J. McNeill, L. Nasdala, M. T. Hutchison, S. Matveev, K. Mather, G. Silversmit, S. Schmitz, B. Vekemans, L. Vincze, Hydrous mantle transition zone indicated by ringwoodite included within diamond. *Nature* **507**, 221–224 (2014).

37. M. J. Walter, S. C. Kohn, D. Araujo, G. P. Bulanova, C. B. Smith, E. Gaillou, J. Wang, A. Steele, S. B. Shirey, Deep mantle cycling of oceanic crust: Evidence from diamonds and their mineral inclusions. *Science* **334**, 54–57 (2011).

38. A. H. Peslier, A. B. Woodland, J. A. Wolff, Fast kimberlite ascent rates estimated from hydrogen diffusion profiles in xenolithic mantle olivines from Southern Africa. *Geochim. Cosmochim. Acta* **72**, 2711–2722 (2008).

39. A. F. Riva, A. D. Rosa, C. Clavel, D. Sifre, O. Mathon, G. Garbarino, S. Pascarelli, Heat distribution in paris–edinburgh press assemblies through finite element simulations. *High Pressure Res.* **38**, 303–324 (2018).

40. W.-Y. Zhou, M. Hao, R. Hrubiak, C. Kenney-Benson, J. S. Zhang, Migration and accumulation of hydrous mantle incipient melt in the Earth's asthenosphere: Constraints from in-situ falling sphere viscometry measurements. *Earth Planet. Sci. Lett.* **641**, 118833 (2024).

41. N. R. Bennett, J. M. Brenan, Controls on the solubility of rhenium in silicate melt: Implications for the osmium isotopic composition of earth's mantle. *Earth Planet. Sci. Lett.* **361**, 320–332 (2013).

42. H. Faxén, Der Widerstand gegen die Bewegung einer starren Kugel in einer zähen Flüssigkeit, die zwischen zwei parallelen ebenen Wänden eingeschlossenist. *Ann. Phys.* **373**, 89–119 (1922).

43. A. D. Maude, End effects in a falling-sphere viscometer. *Br. J. Appl. Phys.* **12**, 293–295 (1961).

44. Y. Kono, C. Kenney-Benson, Y. Shibazaki, C. Park, G. Shen, Y. Wang, High-pressure viscosity of liquid Fe and FeS revisited by falling sphere viscometry using ultrafast X-ray imaging. *Phys. Earth Planet. In.* **241**, 57–64 (2015).

45. M. Brizard, M. Megharfi, E. Mahé, C. Verdier, Design of a high precision falling-ball viscometer. *Rev. Sci. Instrum.* **76**, 025109 (2005).

46. M. Mercier, A. D. Muro, N. Métrich, D. Giordano, O. Belhadj, C. W. Mandeville, Spectroscopic analysis (ftir, raman) of water in mafic and intermediate glasses and glass inclusions. *Geochim. Cosmochim. Acta* **74**, 5641–5656 (2010).

47. M. Gao, C. Wang, H. Xu, J. Zhang, Experiment constraints on orthopyroxene enrichment in the Kaapvaal Craton lithospheric mantle. *Chem. Geol.* **562**, 120046 (2021).

48. F. R. Boyd, N. P. Pokhilenko, D. G. Pearson, S. A. Mertzman, N. V. Sobolev, L. W. Finger, Composition of the Siberian cratonic mantle: Evidence from Udachnaya peridotite xenoliths. *Contrib. Mineral. Petrol.* **128**, 228–246 (1997).

49. E. S. Persikov, P. G. Bukhtiyarov, A. G. Sokol, Viscosity of Haplokimberlitic and basaltic melts at high pressures: Experimental and theoretical studies. *Chem. Geol.* **497**, 54–63 (2018).

50. E. J. Holden, S. Moss, J. K. Russell, M. C. Dentith, An image analysis method to determine crystal size distributions of Olivine in Kimberlite. *Comput. Geosci.* **13**, 255–268 (2009).

51. T. F. D. Nielsen, K. K. Sand, The Majuagaa Kimberlite Dike, Maniitsoq Region, West Greenland: Constraints on an Mg-rich silicocarbonatitic melt composition from groundmass mineralogy and bulk compositions. *Can. Mineral.* **46**, 1043–1061 (2008).
